# Platelet Redox Imbalance in Hypercholesterolemia: A Big Problem for a Small Cell

**DOI:** 10.3390/ijms231911446

**Published:** 2022-09-28

**Authors:** Alessandro Morotti, Cristina Barale, Elena Melchionda, Isabella Russo

**Affiliations:** Department of Clinical and Biological Sciences of the Turin University, Regione Gonzole, 10, I-10043 Orbassano, TO, Italy

**Keywords:** platelet activation, oxidative stress, hypercholesterolemia, proprotein convertase subtilisin/kexin type 9, statins

## Abstract

The imbalance between reactive oxygen species (ROS) synthesis and their scavenging by anti-oxidant defences is the common soil of many disorders, including hypercholesterolemia. Platelets, the smallest blood cells, are deeply involved in the pathophysiology of occlusive arterial thrombi associated with myocardial infarction and stroke. A great deal of evidence shows that both increased intraplatelet ROS synthesis and impaired ROS neutralization are implicated in the thrombotic process. Hypercholesterolemia is recognized as cause of atherosclerosis, cerebro- and cardiovascular disease, and, closely related to this, is the widespread acceptance that it strongly contributes to platelet hyperreactivity via direct oxidized LDL (oxLDL)-platelet membrane interaction via scavenger receptors such as CD36 and signaling pathways including Src family kinases (SFK), mitogen-activated protein kinases (MAPK), and nicotinamide adenine dinucleotide phosphate (NADPH) oxidase. In turn, activated platelets contribute to oxLDL generation, which ends up propagating platelet activation and thrombus formation through a mechanism mediated by oxidative stress. When evaluating the effect of lipid-lowering therapies on thrombogenesis, a large body of evidence shows that the effects of statins and proprotein convertase subtilisin/kexin type 9 inhibitors are not limited to the reduction of LDL-C but also to the down-regulation of platelet reactivity mainly by mechanisms sensitive to intracellular redox balance. In this review, we will focus on the role of oxidative stress-related mechanisms as a cause of platelet hyperreactivity and the pathophysiological link of the pleiotropism of lipid-lowering agents to the beneficial effects on platelet function.

## 1. Introduction

Oxidative stress results from an imbalance between the synthesis of reactive oxygen species (ROS) and their neutralization by anti-oxidants [1] and the role of ROS as a second messenger for intracellular signalling has been well established in several cell lines, including platelets. A great deal of evidence shows that both increased intraplatelet ROS synthesis and impaired ROS neutralization are implicated in the thrombotic process.

A prothrombotic phenotype is associated with a number of metabolic disorders related to dyslipidemia, including metabolic syndrome, diabetes, and atherosclerosis [2,3,4,5,6,7,8]. Low-density lipoprotein cholesterol (LDL-C) is recognized as a key player in the pathogenesis of atherosclerosis, and any effective effort is advocated to reduce LDL-C levels to prevent cerebro- and cardiovascular (CV) risk [2].

Increased platelet reactivity is involved in the pathophysiology of occlusive arterial thrombi associated with myocardial infarction and stroke in dyslipidemia, thus increasing the risk for coronary events and mortality for subjects showing various measures of platelet reactivity [2,9,10,11,12]. A number of markers of platelet activation positively correlate to cholesterol levels [13,14,15] and several studies showed that hyperlipidemia is associated with CV events [16,17,18]. In this context, the imbalance between cellular pro- and anti-oxidant pathways leads to an overproduction of ROS that ends up impairing signallings involved, specifically, in the modulation of platelet response to agonists and inhibitors. Additionally, hypercholesterolemia can also contribute to platelet hyperreactivity via direct oxidized LDL (oxLDL)-platelet membrane interaction via scavenger receptors such as CD36 and signalling pathways including Src family kinases (SFK), mitogen-activated protein kinases (MAPK) and nicotinamide adenine dinucleotide phosphate (NADPH) oxidase [19,20,21,22]. Activated platelets, in turn, can induce the activation of other platelets, increasing their atherogenic effect, and promote LDL-C oxidation through the synthesis of ROS by NADPH oxidase activity, thus propagating platelet activation [23]. If oxLDL is deeply involved in promoting platelet activation, LDL-lowering treatment also affects platelets [14]. Indeed, drugs used as lipid-lowering agents able to lower cholesterol levels show beneficial CV effects because of their ability to affect platelet aggregability, possibly by regulating intraplatelet redox imbalance independently of their hypolipidemic properties [24,25,26].

In this review, we focus on the currently available evidence on the oxidative stress-related mechanisms underlying platelet hyperreactivity in hypercholesterolemia and the pathophysiological link of the pleiotropism of lipid-lowering agents to beneficial effects on platelet function.

## 2. Mechanisms of Platelet Activation: Role of Oxidative Stress

Platelets exert a crucial role in normal hemostasis, whereas pathological platelet reactivity is correlated with cardiovascular events. The underlying cause in cardiovascular disorders includes rupture of the plaque with subsequent platelet adhesion, activation, release of granule content, and eventual thrombosis [27,28,29,30].

**Figure 1:**
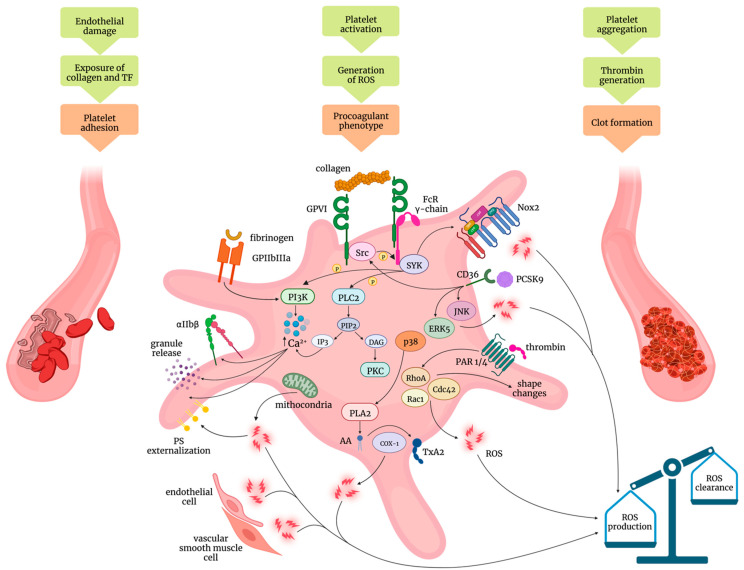
Platelet redox imbalance leading to a prothrombotic state. Major signalling pathways involved in platelet activation and increased ROS production. The imbalance between ROS production and ROS clearance causes oxidative stress and contributes to the generation of a prothrombotic state. Abbreviations: TF: tissue factor; ROS: reactive oxygen species; GPIIbIIIa: glycoprotein IIb/IIIa; PI3K: phosphoinositide 3-kinase; GPVI: glycoprotein VI; FcR γ-chain: Fc receptor γ-chain; SYK: spleen tyrosine kinase; PLC2: phospholipase C2; PIP2: phosphatidylinositol (4,5) bisphosphate; IP3: inositol trisphosphate; DAG: diacylglycerol; PKC: protein kinase C; Nox2: NADPH oxidase 2; PCSK9: proprotein convertase subtilisin/kexin type 9; CD36: cluster of differentiation 36; JNK: c-Jun N-terminal kinase; ERK5 Extracellular signal-regulated kinase 5; PLA2: phospholipase A2; AA: arachidonic acid; COX-1: cyclooxygenase 1; TxA2: thromboxane A2; PAR1/4: protease-activated receptor 1/4; RhoA: Ras homolog family member A; CDC42: cell division cycle 42; Rac1: Ras-related C3 botulinum toxin substrate 1; αIIbβ: integrin α IIb β; PS: phosphatidylserine.

After interaction with the exposed collagen and tissue factor, following vessel damage, platelets undergo biochemical events, eventually leading to platelet activation and clot formation, events including platelet adhesion to the site of injury, platelet spreading and aggregation, as well as a shift of a subset of platelets to procoagulant phenotype (Figure 1).

In hemostasis these events are necessary to prevent blood loss, whereas “unchecked” mechanisms leading to occlusion of the blood vessel are associated with thrombotic complications. Platelet adhesion molecules allow cell adhesion to the exposed extracellular matrix and, upon platelet activation at the site of injury, inside-out signaling activates the complex permitting binding to proteins such as von Willebrand factor (VWF) produced by endothelial cells. Platelet-derived ADP and thromboxane activate non-adhered platelets via their glycoproteins (GP)IIb/IIIa receptors, allowing these platelets to participate in platelet aggregation [31]. ROS are critical mediators behind platelet activation induced by stimuli acting by different pathways. Indeed, a variety of ligands are able to increase platelet generation of ROS that, in turn, can activate platelet surface receptors and induce the release of contents of preformed intracellular granules.

G-protein coupled receptors represent the two main subgroups of receptors that mediate the agonist-induced activation of platelets (Figure 1). GPVI is expressed in both platelets and megakaryocytes and its crosslinking to collagen activates Src family kinase-mediated phosphorylation of the immunoreceptor tyrosine-based activation motif (ITAM) on Fc receptors (FcR) γ-chain and the recruitment of the non-receptor tyrosine kinase spleen tyrosine kinase (SYK) [32,33]. Activation of phospholipase Cγ2 (PLCγ2) and protein kinase C (PKC) precedes the cytosolic calcium mobilization, essential for integrin αIIbβ activation, platelet granule release reaction, and the expression of procoagulant phosphatidylserine [34,35,36]. Indeed, GPVI may participate in other platelet functions beyond its classic role as a platelet adhesion receptor. It was shown to be crucial in inducing platelet aggregation of pre-formed thrombus also in the absence of thrombin [37].

A large body of evidence underlines the importance of ROS-dependent pathways in platelet adhesion and activation [38,39,40]. GPVI-ITAM signaling is involved in promoting ROS production through the activation of NADPH oxidase, a multi-subunit complex on the cell membrane recognized as a major ROS–generating enzyme in the vasculature [41] with a specific role in the mechanism of intracellular signalling activation by transferring reducing equivalents from NADPH to molecular oxygen [42,43,44]. In platelets, ROS from NADPH oxidase and other sources are important modulators of procoagulant phosphatidylserine (PS) externalization [45,46], and studies suggest their involvement also as a step for the subsequent platelet activation. Indeed, inhibiting NADPH oxidase by using the nonspecific inhibitor apocynin or diphenyleneiodonium decreased both ROS generation and platelet activation [38,44,47,48]. On the other hand, no genotype-dependent differences were found in detecting ROS levels as well as in the adhesion, activation, and aggregation of platelets from the NADPH oxidase (Nox2)-deficient animal model or in human patients with no functional NADPH oxidase [49]. The transmembrane catalytic subunit of NADPH oxidase Nox2 (gp91phox), responsible for the generation of large amounts of ROS catalytic subunit of NADPH oxidase, has been suggested to play a key role in mediating platelet ROS production and activation based on observations from the platelets of patients with chronic granulomatous disease, an X-linked disease related to mutations in the gene encoding Nox2 *CYBB* [50]. Indeed, platelets from these patients showed reduced agonist-induced ROS production but normal platelet aggregation [38,47,48], indicating that major platelet functions are not influenced by the lack of Nox2.

However, others indicate that NADPH oxidase is an agonist-specific functional source of platelet ROS production [43,51,52,53,54], and thus the exact role of NADPH oxidase in platelet activation and ROS generation needs further investigation. Although the role of ROS on platelet adhesion and aggregation is unequivocal, it has been suggested that alternative sources of ROS mediate the functional responses of platelets to stimuli, rather than Nox2 NADPH oxidase [49]. This is in agreement with other papers reporting mitochondrial ROS involvement in platelet activation in different clinical settings [55,56], as well as stimulatory effects on platelet activation which were observed for nitric oxide synthase (NOS) [57,58] and other oxidant proteins such as p66Shc [59].

A number of soluble agonists released by damaged cells (i.e., ADP) or produced in the course of inflammation (i.e., platelet-activating factor (PAF)), coagulation (i.e., thrombin) or secreted by stimulated platelets (i.e., thromboxane A2 (TXA2), ADP, serotonin) activate platelets via G-protein-coupled receptors, a family of seven-transmembrane domain receptors that transduce signals through heterotrimeric G proteins [60]. For platelet adhesion and thrombus formation processes, platelet cytoskeletal reorganization is an essential step. The Ras homologous (Rho) family of GTPase members Ras-related C3 botulinum toxin substrate (Rac)1, cell division cycle (Cdc)42 and RhoA have been recognized not only as major components of the intracellular signalling network crucial for morphological dynamics and shape change, but also in the ROS modulating platelet function. Indeed, platelet RhoGTPases can orchestrate the formation of filopodia and lamellipodia to strongly increase the platelet surface upon activation and oxidative processes in an interconnected manner to regulate intracellular signalling networks underlying platelet activation leading to thrombus generation.

The serine protease thrombin is a potent platelet activator and its generation comes from proteolytic activation of its zymogen prothrombin by the prothrombinase complex assembled downstream of intrinsic and extrinsic coagulation pathways. It promotes the activation of platelets by cleaving platelet protease-activated receptors (PARs) [61,62]. In human platelets, thrombin mainly stimulates the Gαq-protein-coupled receptors PAR1/4. PAR1 operates at low concentrations of thrombin, whereas PAR4 is activated only in the presence of a higher concentration of this agonist [62]. The downstream activation of phospholipase Cβ (PLCβ) leads to the formation of the second messengers inositol trisphosphate (IP_3_) and diacylglycerol (DAG) [63], followed by the increase of Ca^2+^ endoplasmic reticular release and PKC activation, respectively [64,65]. The responses to thrombin stimulation will be shape change, granule secretion and aggregation. Platelet shape change is regulated by both calcium-dependent and independent mechanisms [66] occurring through G_q_ and G_12/13_ pathways, respectively. G_12/13_ pathways regulate calcium-independent and RhoA-dependent responses [67], and the RhoA-p160^ROCK^ pathway is involved in G_12/13_-mediated dense granule secretion and platelet shape change [68,69,70].

There is a link between platelet activation regulated by endogenous ROS induced by some agonists (e.g., thrombin, thrombin receptor activator peptide (TRAP)-6 and U46619) and mitochondria dysfunction [48,71,72]. Mitochondria are relevant sources of ROS [73,74,75], and the coupling between redox reactions and ATP synthesis also leads to the prothrombotic function of platelets for the formation of procoagulant platelets.

Upon activation, platelets lose part of their contents to the environment in different ways, i.e., by granule secretion, formation of extracellular vesicles (microparticle), and receptor shedding [62]. A number of platelet agonists evoke the release of TXA2, formed from arachidonate conversion by the activity of the cyclooxygenase-1 (COX-1)-thromboxane synthase complex [28,76], and the increased COX-1 activity contributes to generating ROS [27], thus enhancing the recruitment of platelets to a growing thrombus [77].

An important contribution to platelet activation is made by ROS released from other sources (e.g., endothelial and vascular smooth muscle cells), especially for their ability to scavenge nitric oxide (NO) [78] and regulate the redox-sensitive ectonucleotidases on the platelet membrane [39]. Furthermore, in the presence of higher ROS production an enhanced lipid peroxidation of circulating LDL or cell-membrane phospholipids may occur with the consequent generation of the prostaglandin isomers F2-isoprostanes [79,80] by mechanisms catalyzed by free radicals. F_2_-isoprostanes are able to amplify activation and the adhesive reactions of platelets in response to other agonists [81].

Experiments carried out on animal knock-out for antioxidant enzymes as well as data from clinical studies using drugs able to interfere in ROS production confirmed [82,83,84] the role of increased intracellular oxidative stress on platelet activation.

## 3. Platelet Reactivity in Dyslipidemia

Dyslipidemia is a relevant risk factor for the development of atherosclerosis due to the chronic accumulation of lipid-rich plaque in arteries [85]. However, dyslipidemia is supposed to increase CV risk depending as much on its long-term effects on atherogenesis as on its influence on thrombogenesis [2]. Alterations of the lipid profile are associated with both increased oxidative stress and the generation of oxidized lipids, which exert an important role in promoting atherogenesis through their influence on endothelial and vascular smooth muscle cells, the formation of lipid-laden foam cells [86] and platelet hyperreactivity [6,87,88,89]. In turn, activated platelets contribute to oxLDL generation, which ends up propagating platelet activation and thrombus formation through a mechanism mediated by oxidative stress. Specifically, the increased activity of NOX-2 is implicated in the enzymatic pathway leading to ROS synthesis by which platelets propagate the oxidation of lipoproteins [21,23].

Biologically active oxidized phospholipids are deeply involved in initiating and modulating a number of cellular events attributed to atherogenic processes [90]. The link between dyslipidemia and platelet hyperactivation is well-supported by in vitro studies of platelet reactivity and major adverse cardiac events in patients with coronary artery disease (CAD) [3,91,92]. Platelets from individuals affected by CAD and/or familial hypercholesterolemia show increased reactivity when activated by classic platelet agonists.

Furthermore, in vivo studies conducted in atherogenic mice (*apoE* or *ldlr* null mice undergone high fat diet) showed a prothrombotic phenotype after that thrombosis was chemically induced by the oxidant ferric chloride [91] as such, understanding the pathways leading to platelet activation in dyslipidemia has aroused much interest also to prevent thrombotic events. The first correlation between dyslipidemia and elevated platelet activation was found by Carvalho in patients affected by hypercholesterolemia [3], then confirmed also in dyslipidemic animal models [93] and even in the presence of mild hyperlipidemia [94]. Indeed, since the first observation, our knowledge of this topic including the associated mechanisms has dramatically increased. In addition to clinical studies, a large number of in vitro studies have been performed by exploring the effects of different laboratory oxLDL leading to find a plethora of signalling events in platelets activated by these atherogenic lipoproteins. It has been shown that oxLDL can interfere with cardinal platelet function such as adhesion [95,96,97,98], secretion [19,95,99,100,101], ROS generation [20,21,97], and microvesicles release, triggering a procoagulant phenotype with phosphatidylserine exposure [102], by acting synergistically with agonists, aggregation [103,104,105] even if, in other in vitro studies, low oxLDL levels seem attenuate platelet function and aggregation [106,107,108,109]. These contradictory findings could depend on the heterogeneous nature of oxLDL, such as the oxidized domain and extent of oxidation [110]. Actually, in vitro experiments demonstrate that the same LDL sample differently oxidized produces different products with distinct functional responses [111,112,113]. Thus, the presence of different scavenger receptors binding to oxLDL combined with the heterogenous composition of oxidized particles may justify the different responses of platelets to these lipoproteins even if, generally, they are linked to signallings driving to platelet activation.

**Figure 2:**
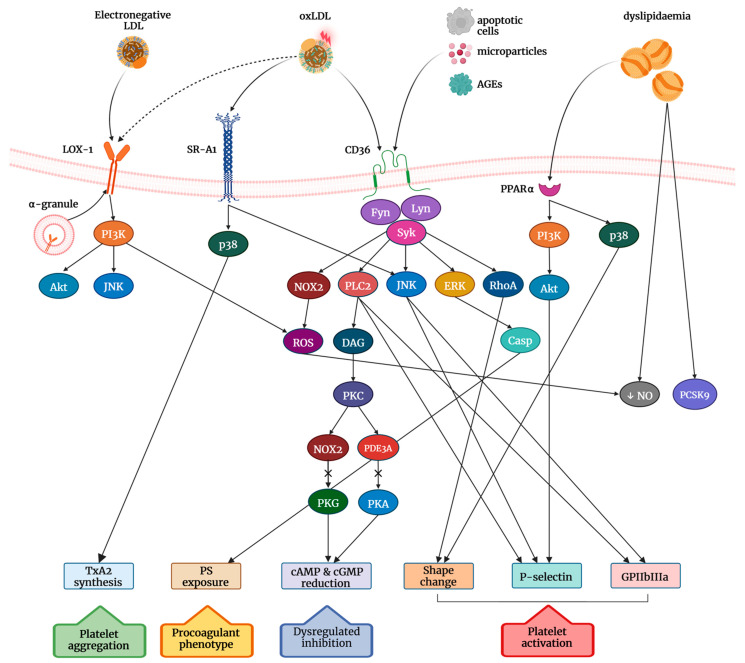
Signaling pathways enhanced by oxLDL binding to platelet SRs and PPARα expression in dyslipidaemia. Abbreviations: LDL: low density lipoprotein; LOX-1: lectin-like oxidized low-density lipoprotein receptor-1; PI3K: phosphoinositide 3-kinase; Akt: Protein kinase B; JNK: c-Jun N-terminal kinase; oxLDL: oxidized LDL; SR-A1: scavenger receptor A1; TxA2: thromboxane A2; CD36: cluster of differentiation 36; AGEs: advanced glycation end products; SYK: spleen tyrosine kinase; Nox2: NADPH oxidase 2; PLC2: phospholipase C2; ERK Extracellular signal-regulated kinase; RhoA: Ras homolog family member A; ROS: reactive oxygen species; DAG: diacylglycerol; Casp: caspase; PKC: protein kinase C; PDE3A: phosphodiesterase 3A; PKG protein kinase G; PKA protein kinase A; cAMP: cyclic adenosine monophosphate; cGMP: cyclic guanosine monophosphate; PPARα: peroxisome proliferator-activated receptor alpha; NO: nitric oxide; PCSK9: proprotein convertase subtilisin/kexin type 9; GPIIbIIIa: glycoprotein IIb/IIIa; PS: phosphatidylserine.

An emerging role in the prothrombotic tendency related to dyslipidemia is the activation of peroxisome proliferator-activated receptor alpha (PPARα) signalling supporting platelet activity [114]. PPARα is involved in the regulation of lipid metabolism, and hyperlipidemia per se is caused by the increased PPARα expression in megakaryocytes but not in platelets [114]. This fact indicates a role for PPARα in bridging the genetic effect of impaired hemostasis (aggregation, adenosine 5′-triphosphate (ATP) secretion, spreading) and thrombosis, with PPARα required for platelet activation and thrombus formation. The positive correlation between the extent of platelet reactivity and PPARα expression both in hyperlipidemic mice and patients with hyperlipidemia supports the role of PPARα in mediating platelet activation. The effect of PPARα on platelets involves p38MAPK/Akt and downstream ROS generation controlled by fatty acid β-oxidation, and NADPH oxidase pathways activated in dyslipidemic conditions of high-fat diet or exposure to oxLDL [114]. Therefore, the PPARα/p38/ROS/Akt axis is supposed to act as a central gatekeeper for platelet activation eventually employed by major platelet receptors such as G-protein-coupled receptors and the scavenger receptor CD36 (Figure 2).

## 4. Role of Scavenger Receptors in the OxLDL-Induced Signalling Transduction

The best characterized receptor for oxLDL is the scavenger receptor CD36, also known as platelet GP IV or fatty acid translocase [115,116], expressed up to 20,000 copies per platelet and associated with the Src family members Fyn, Lyn, and Yes [117] (Figure 2). Identified as a platelet surface glycoprotein initially thought to function as a collagen receptor, CD36 acts as a circulating sensor of chronic disorders being able to recognize ligands such as oxLDL, apoptotic cells, advanced glycated proteins, and microparticles. Specifically, CD36, as a sensor of oxidative stress and player of platelet response, links hyperlipidemic conditions to prothrombotic state [91]. The finding that platelets from *Cd36*^−/−^ mice as compared to wild-type mice on a normal chow diet do not reduce their response to agonists restricts the role of CD36 in thrombosis only if in the presence of a dyslipidemic milieu, where the increased oxidative stress can generate structurally defined endogenous ligands [91,118]. The signalling pathways influenced by CD36 in platelets include both the synergizing effect in pathways activated by common agonists and, indirectly, the attenuation of the activation of the inhibitory pathways adenosine 3′,5′-cyclic monophosphate (cAMP)/protein kinase A (PKA) [119] and guanosine 3′,5′-cyclic monophosphate cGMP/PKG (Figure 2) [21]. Src family kinases connect CD36 to platelet activation through the activation of MAPKs c-Jun N-terminal kinase (JNK) [19,120] and extracellular signal-regulated kinase (ERK) [20], PKC, PLCγ2, and NADPH oxidase with subsequent production of superoxide radical anion [22,72,121,122].

The NADPH oxidase-mediated ROS generation by CD36 induces platelet ERK-5 activation that promotes aggregation and the procoagulant phenotype [123], the last-one due to increased externalization of PS mediated by GPVI signalling activation. CD36 and ERK-5 mediated PS externalization increases factor tenase and prothrombinase activation, thus promoting fibrin deposition. These mechanisms are responsible, at least in part, for the increased thrombin formation and procoagulant function mediated by platelets in dyslipidemia [123,124].

The atherogenic oxidized choline glycerophospholipids oxPC_CD36_ with high affinity for CD36 binding, present in vivo at sites of enhanced oxidative stress [125] and markedly increased in hyperlipidemic plasma, is linked to enhanced platelet reactivity and the prothrombotic phenotype [91].

An additional scavenger receptor that regulates platelet reactivity in dyslipidemia is scavenger receptor A-1 (SRA-1), which induces platelet activation through a p38 MAPK-dependent mechanism [126]. Platelet activation by SRA-1 seems to involve CD36 because in the presence of CD36-deficient mouse platelets or CD36 inhibitors the platelet spreading on collagen normally induced by oxidized lipids through SRA-1 activation is blunted. Indeed, the various degree of oxidation of lipids can differently induce the activation of CD36 compared to SRA-1 (Figure 2).

In dyslipidemia, platelet hyperreactivity, strongly and independently associated with thrombotic events [91,127,128,129,130,131], is characterized by the enhanced expression of activated GPIIbIIIa, P-selectin, and, as already mentioned, higher platelet aggregation to agonists [14,15,132]. Mechanistically, some studies have shown that increased levels of ROS and oxidation reactions are a cause of hyperaggregability and activation [56,133,134,135], even though in the association of dyslipidemia with CV diseases a role is played by the effects per se of plasma oxidized lipids on platelet function [136]. Actually, platelet membrane exposure to cholesterol accumulation can cause multiple alterations of membrane morphology and function, also depending on the heterogeneous nature of lipoproteins. In their native form, LDL particles are not able to increase platelet aggregation, whereas the oxidative modifications of LDL make LDL an independent platelet activator also inducing platelet aggregation in the absence of naturally occurring agonists [137].

Lectin-like oxLDL scavenger receptor-1 (LOX-1), a type-II membrane protein belonging to the C-type lectin family and consisting of four domains, is a major receptor for oxLDL uptake in vascular cells and plays a pivotal role in stimulating ROS production [138]. In platelets, LOX-1 is located in alpha granules and found on the cell surface upon platelet activation [139], thus indicating that LOX-1 may play a role in amplifying oxLDL effects secondary to the initial activation of platelets. In other words, it may suggest that the increased expression of LOX-1 on the platelet surface associated with disease potentiates the activation of preactivated platelets facilitating a more pronounced response to circulating oxLDL. However, the absence of LOX-1 expression in stable conditions with resting platelets makes it difficult to establish the exact role of LOX-1. Conversely, CD36 is present on the plasma membrane both in resting and activated platelets. LOX-1 differs from CD36 also in binding oxLDL because it recognizes both mildly oxidized phospholipids and delipidated oxLDL [110]. Once on the platelet surface, LOX-1 is able to bind electronegative LDL driving the stimulation of signalling events including PI3K and MAPK/JNK which increase of p-selectin and GPIIb/IIIa expression [110]. Electronegative LDLs include mildly oxidized LDL with highly electronegative fractions that increase platelet aggregation to ADP, p-selectin and integrin expression.

As is known, the loss and/or impaired effects of NO on platelets can be responsible for increased platelet activation and in disorders, including hypercholesterolemia, the reduced bioavailability of NO due to the increased oxidative stress becomes an important determinant for platelet hyperactivation [140]. Indeed, in hypercholesterolemia with increased levels of LDL combined with the higher generation of oxLDL, platelet hyperactivation could also depend not only on reduced bioavailability but also on decreased sensitivity to NO-related pathways [141,142,143,144]. In patients affected by primary hypercholesterolemia, the greater tendency of platelets to aggregate in response to the naturally occurring agonists such as ADP, collagen, and arachidonic acid (AA) is associated with higher ROS levels, the reduced antiaggregating effect of NO, and higher PI3K/Akt and MAPK/ERK-2 pathway activation. Moreover, platelets also show a resistance to the NO-mediated inhibitory effects of the incretin hormone glucagon-like peptide 1(GLP-1) [141,145]. In this phenomenon, a role could be played by oxLDL’s ability to generate Nox2-derived ROS through a CD36-PKC pathway followed by the inhibition of cGMP signalling [21], a NO-mediated pathway that, if overcome, leads to platelet activation. Specifically, with regard to the NO-mediated pathway, circulating platelets are kept in a quiescent state for NO’s ability to stimulate the intracellular receptor guanylyl cyclase (GC) that, via the second messenger cGMP, inhibits platelet activation by stimulating protein kinase G. A reduced activation of the NO/cGMP/PKG pathway increases platelet sensitivity to agonists contributing to platelet hyperactivation [146,147].

Patients with hypercholesterolemia show increased secretion of TXA_2_, the superoxide anion, and higher circulating levels of platelet activation markers including sP-selectin, PF-4, sCD-40L, and β-thromboglobulin [14,15,132]. Many of these platelet alterations are corrected by drugs aimed at lowering cholesterol levels such as statins and anti-proprotein convertase subtilisin/kexin type 9 (PCSK9) antibodies [14,141]. Markers of oxidative stress, endothelial dysfunction, subclinical inflammation, and platelet activation as well as platelet sensitivity to the inhibitory effects of NO [141] and aspirin [14] were significantly improved by treatment with lipid-lowering drugs [14,15,148,149,150,151,152].

## 5. Role of PCSK9 on Platelet Activation in Hypercholesterolemia

PCSK9 is a serine protease, expressed in many tissues and cell types [153], and is synthetized as a soluble zymogen enzymatically activated by itself. PCSK9 is known for its non-enzymatic and critical ability to promote the degradation of LDL receptor (LDLR) [154,155]. In light of this, the catalytic activity of PCSK9 is not required for its functional role on LDLR cycling [156]. The pivotal role of PCSK9 is the regulation of cholesterol homeostasis. In the absence of PCSK9, the complex LDLR-LDL-C is internalized and dissociated, with LDL-C directed to degradation in lysosomes and LDLR recycled to the cell surface. The recycled expression of LDLR promotes further LDL uptake from blood with the subsequent reduction of circulating LDL levels. On the contrary, LDLR binding to PCSK9 prevents the recycling of LDLRs, thereby preventing the ingestion of circulating LDL particles and consequently leading to LDL elevation in the blood [157]. An intriguing hypothesis suggests that platelets, when activated by LDL, become an additional source of PCSK9, thereby increasing LDL plasma levels which in turn further stimulates platelets to release PCSK9 [158], even though direct evidence of the ability of platelets to affect the hepatic metabolism is not known. However, one can suppose that, at least in liver circulation, the increased concentration of PCSK9 at the level of the hepatic microenvironment may affect LDL recycling with effects on inflammation and the atherogenesis process.

PCSK9 plays also a role in lipid-independent reactions implicated in the process of atherothrombosis and thrombo-inflammation. This may be a relevant aspect in the highly efficient ability of PCSK9 inhibitors to reduce CV events.

PCSK9 positively modulates platelet function [159], and PCSK9 inhibitors reduce platelet reactivity [15]. The role of PCSK9 in platelet reactivity also comes from studies on experimental animal models with the loss of PCSK9; a significant reduction in the expression of the platelet activation markers glycoprotein IIb/IIIa and P-selectin was observed if compared with wild-type ones [159]. The association of hypercholesterolemia to platelet activation in CVD patients has been established [91,111] and, in this context, increased levels of PCSK9 may be responsible for platelet activation because of the impairment of lipoprotein clearance and the regulatory effects on platelets by native LDL and oxLDL. Plasma membrane exposure to excessive cholesterol can alter membrane structure with sequelae on cell signaling. Indeed, LDL per se is not able to induce platelet aggregation but enhances platelet reactivity to agonists while, if oxidized, LDL can induce platelet aggregation also in the absence of aggregants [137].

Mechanism studies showed that PCSK9 activates platelets by binding to membrane CD36 (Figure 1), thus activating Src kinase and MAPK-ERK 5 and JNK, leading to the generation of ROS, and the activation of p38MAPK/cytosolic phospholipase (PL)A2/cyclooxygenase-1 (COX-1)/TXA_2_ signalling pathways [160]. Experiments conducted in CD36 knockout mice confirmed the role of CD36 in promoting platelet activation by PCSK9 [160]. As recently demonstrated, activated platelets release stored PCSK9 [158], a phenomenon enhanced by platelet exposure to LDL-C with effects on aggregation and thrombus formation under flow dynamic conditions.

## 6. Current Hypolipidemic Drugs with Effects on the Redox Balance of Platelets

### Statins

Statins, recommended both in the primary and secondary prevention of CVDs, are the largest single class of cholesterol-lowering drugs and are still now, after 30 years, the first-line treatment for the prevention of CAD and atherosclerosis [161]. Statins mainly act by reducing cholesterol synthesis in the liver through the inhibition of the activity of 3-hydroxymethyl-3-methylglutaryl coenzyme A (HMG-CoA) reductase [162]. The reduction of intracellular cholesterol leads to an increase in the expression of LDLR on the cell surface and the uptake of LDL from the blood. As a result, the plasma levels of Apolipoprotein B (ApoB) containing lipoproteins decrease [161,162].

Indeed, it has been widely recognized that benefits from statins are dependent not only on their lipid-lowering properties but also on their pleiotropic effects [163,164]. In addition to their anti-inflammatory action, the anti-oxidative and antithrombotic effects of statins, including those also on endothelial function [165,166], have been reported in numerous studies, thus contributing to explain the beneficial effects of statins in reducing CVD [167,168,169,170,171,172,173,174,175,176]. On the other hand, in comparison with statin continuation, especially in older people taking long-term statin treatment both in primary and secondary prevention, statin discontinuation has been associated with a significant increase in the risk of major adverse CV events [177].

**Figure 3:**
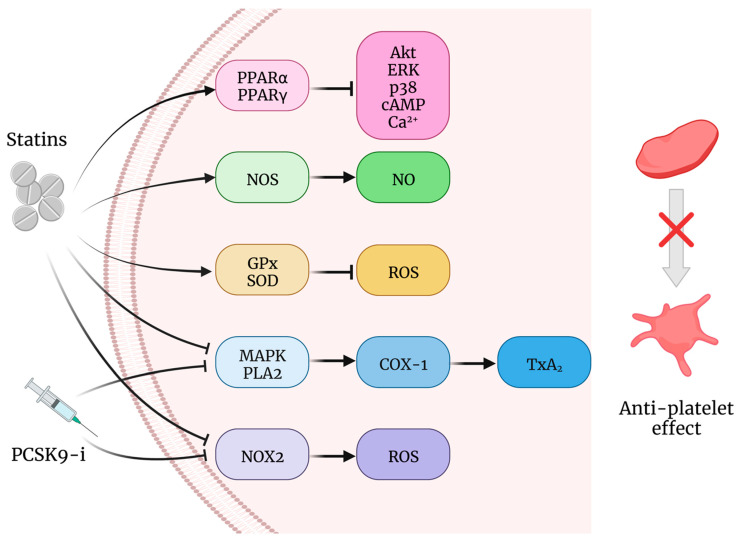
Effects of statins and PCSK9 inhibitors on platelet redox balance. Abbreviation: PCSK9-i: PCSK9 inhibitors; PPARα: peroxisome proliferator-activated receptor alpha; PPARγ: peroxisome proliferator-activated receptor gamma; NOS: nitric oxide synthase; GPx: glutathione peroxidase; SOD: superoxide dismutase; MAPK: mitogen-activated protein kinase; PLA2: phospholipase A2; Nox2: NADPH oxidase 2; Akt: Protein kinase B; ERK Extracellular signal-regulated kinase; cAMP: cyclic adenosine monophosphate; NO: nitric oxide; ROS: reactive oxygen species; COX-1: cyclooxygenase 1; TxA2: thromboxane A2.

Taking into account the concept that statins influence platelet function, it is not surprising that a condition of platelet hyperactivation may occur after statin discontinuation [178]. This finding together with the loss of beneficial effects of statins on endothelial function can contribute to account for an increased hypercoagulable state, thrombus formation, and cardiovascular events rate after statin discontinuation [177]. Some of the antiplatelet effects of statins could be explained by mechanisms summarized in Figure 3.

These include the reduction of intracellular calcium and TXA2 concentrations as a consequence of the inhibition of platelet PLA2 phosphorylation and MAPK pathway activation [179,180,181,182]. Furthermore, the modulation of PPAR alpha and PPAR gamma pathways induced by statins also mediate the activity of molecules such as Akt, cAMP, ERK, p38, and MAPK as well as the concentration of cytoplasmic Ca^2+^, resulting in a reduction of platelet activity [164,183,184].

The downregulation of COX-1 and upregulation of nitric oxide (NO) synthetase activity have been reported to mediate some of the antiplatelet effects of statins [26,185,186]. Indeed, an important issue was to establish whether the modulation of oxidative stress could influence platelet reactivity. Studies on this topic showed that statins can reduce platelet ROS production, thus justifying some of the benefits of statin therapy on platelet function related not only to their lipid-lowering efficacy but also to their antioxidant effects. Indeed, a large body of evidence from studies conducted in hypercholesterolemia [14,187,188,189,190], metabolic syndrome [191], diabetes mellitus [192] or in patients with established atherosclerosis [193,194] clearly show that statins affect platelet activity [14,193,194,195]. Nevertheless, it has been observed that statins can directly interfere with platelet function independently from their action in lipid metabolism. Both in vivo short time exposure after statin ingestion and in vitro statin incubation in platelet samples have demonstrated that statins modify the platelet expression of activation markers and aggregation to agonists. In light of this, it has been proposed that statins can exert both early anti-oxidant non-lowering dependent and late lowering-dependent antiplatelet effects [26]. In their acute effects a crucial role would be played by the statin’s ability to suppress NADPH oxidase activity with the reduction of isoprostane synthesis [48,171]. Conversely, the late effects would be closely associated with the circulating LDL decrease and triggered by the down-regulation of PLA2 with resulting decreased production of TXA2 [26,196]. Specifically, an antioxidant effect has been found for atorvastatin [26,179], simvastatin [14], and rosuvastatin [197]. Nonetheless, we should consider that hypercholesterolemic statins such as simvastatin and atorvastatin increase long-chain polyunsaturated fatty acids with enhanced AA synthesis from endogenous linoleic acid [198,199,200]. Given that AA is the precursor of TXA2 via COX-1 activity, an increase in AA availability can induce an increased production of TXA2, which is a strong aggregating factor. However, AA is also the substrate of anti-inflammatory pathways involving, for example, the lipoxygenase which modulates lipoxins [201,202]. Thus, increased levels of AA do not necessarily mean deleterious effects on the CV system, even though it is plausible to suppose that they could attenuate some antithrombotic effects of statins.

Although statins remain the first-line treatment for the prevention of primary and secondary CV events in hypercholesterolemia, a percentage of patients who either do not reach the predefined target of LDL concentrations or are intolerant to statins need to be treated with different approaches or implementations.

Statin treatment has also been reported to exert protective effects against oxidative stress for their ability to significantly enhance the circulating levels of the antioxidant enzymes GPx and superoxide dismutase (SOD) (Figure 3). Nevertheless, the association between the antioxidant effects of statin and protection against CVD needs to be well established [203].

## 7. PCSK9 Inhibitors

Given the pivotal role of PCSK9 in regulating cholesterol homeostasis and atherothrombotic processes, the inhibition of PCSK9 has become an effective therapeutic tool to correct the lipid profile and address the unmet clinical needs of achieving the LDL-C level goals for patients with a high CV risk [204].

The anti-PCSK9 monoclonal antibodies (mAbs) alirocumab and evolocumab are PCSK9 inhibitors binding circulating PCSK9 and preventing PCSK9 binding to the LDLR. As a result, treatment with these anti-PCSK9 mAbs increases LDLR expression in the cell membrane, thereby decreasing circulating LDL-c levels [205]. Alirocumab and evolocumab, in addition to maximum tolerated statin therapy, are two human anti-PCSK9 monoclonal antibodies used in patients with familial and non-familial hypercholesterolemia or mixed dyslipidaemia who do not reach their therapeutic goals even with the highest tolerated doses of statins [206,207,208,209]. Data from randomized controlled trials showed a clinical benefit in terms of a dramatic decrease of LDL-C and a positive impact on CV events for both evolocumab [210] and alirocumab [211], even more than for statins, despite the same LDL-C reduction [212]. Indeed, from a clinical point of view, PCSK9 inhibition induces a dramatic reduction of circulating LDL levels, ranging from 50–60% [213,214] and, importantly, a reduction of about 15% in the risk for major adverse cardiovascular events (MACE) (death from CV causes, myocardial infarction, stroke, hospitalization for unstable angina, or coronary revascularization) [210,211], thereby indicating a deep involvement of PCSK9 on the thrombotic process in acute coronary events.

The reduction of cardiovascular morbidity and mortality after inhibiting PCSK9 action could be ascribed not only to the lipid-lowering properties of alirocumab and evolocumab but also, similarly to statins, to their pleiotropic effects [215,216,217]. Among those, it has been shown that PCSK9 inhibitors may directly influence the hemostatic system there being a strong correlation between circulating PCSK9 concentrations and platelet hyperreactivity [15,218,219]. Patients with primary hypercholesterolemia treated up to 12 months with anti-PCSK9 mAbs not only decreased their LDL-C concentration by at least 50–60% but also showed a significant reduction of platelet activation accompanied by the improvement of platelet sensitivity to the inhibitory effects of aspirin [15]. Indeed, on-aspirin hypercholesterolemics, the PCSK9 inhibition decreased the AA-triggered platelet aggregation, reaching a percentage of aggregation values lower than 20%, thus suggesting an improvement in the suppression of the AA-induced TXB2 synthesis. Interestingly, in a further study, after anti-PCSK9 mAbs treatment, these subjects also showed a significant reduction of the LDL oxidation combined with the lower activation of NOX2 in platelets. Collectively, the hypothesis is that, in the presence of PCSK9 inhibitors, the reduction of oxLDL might be due to both a lower availability of LDL substrate and its oxidation mediated by NOX2 activation via CD36 and LOX-1 [219]. Therefore, the effects of PCSK9 inhibition on platelets would be mainly supported by the modulation of redox status in favor of reduced oxidative stress (Figure 3).

New other gene-editing technologies targeting PCSK9 are becoming promising tools for the management of hypercholesterolemia [220,221]. Among those, inclisiran, a long-acting, short-chain small interfering RNA (siRNA) directed against the PCSK9 protein, blunts the natural pathway of PCSK9 gene expression. As a result, the degradation of PCSK9 mRNA reduces the synthesis and secretion of PCSK9. Due to this mechanism, inclisiran causes a reduction in both intracellular and extracellular PCSK9 protein levels with a relevant reduction in plasma LDL-C concentrations for a long time [222,223,224]. So far, inclisiran has been evaluated in preclinical studies and phase 1 and phase 2 clinical trials, and is currently being evaluated in phase III clinical trials. The ORION program, including completed and ongoing clinical trials conceived to investigate the safety and efficacy of inclisiran and its ability to improve cardiovascular outcomes, has confirmed the robust LDL-C lowering properties of inclisiran (284 mg) in the long-term with a reduction of plasma PCSK9 levels by approximately 80% [225,226] and LDL-C levels by approximately 50% compared to placebo. Although at the moment no data from the literature are available, one can suppose that inclisiran’s effects on platelet oxidative balance are like the other PCSK9 inhibitors already tested. Further studies in this direction are required to confirm this aspect.

An additional benefit of inclisiran is a less frequent dosing regimen because its subcutaneous administration is required only once every three to six months, thus improving the compliance compared to daily oral agents.

## 8. Antioxidant Effects on Platelets in Hypercholesterolemia

As mentioned, the redox balance depends on the ROS production and action of non-enzymatic or enzymatic antioxidants. Hypercholesterolemia determines increased ROS levels not only by increasing the activity of redox enzymes, including NADPH oxidase, xanthine oxidase, uncoupled NOS, and the stimulation of the mitochondrial electron-transport chain [227,228], but also by impairing the protective function of antioxidant enzymes that include glutathione peroxidase (GSH-Px), SOD, and catalase [229,230]. GSH-Px catalyses the reduction of H_2_O_2_, to H_2_O and GPHx1 is the most common isoform in human [231]. SOD isoforms (SOD1-3) catalyse the transformation of the superoxide anion O_2_^−^_,_ into O_2_ and H_2_O_2_ [232], whereas catalase promotes the dismutation of H_2_O_2_ into O_2_ and H_2_O [233]. An imbalance of redox status corresponding to the lower activity of the oxidant scavenger SOD correlated with high on-aspirin platelet reactivity was found in patients with primary hypercholesterolemia [234]. Furthermore, the negative correlation between SOD and 11-dhTXB2 in the same subjects underlines the role of oxidant species, such as hydrogen peroxide, as a stimulus for the production of platelet thromboxane and supports the evidence that the deficiency of antioxidant potential causes platelet activation and thrombus formation [84].

For the important role of oxidative stress in pathways involved in platelet activation, several studies have evaluated the putative association between dietary antioxidant nutrients in dyslipidemia and protection against cardiovascular disease [235] and, specifically, the effects of antioxidants on platelet function.

Some plant-derived bioactive flavonoids provide health benefits with regard to the risk for CV events [236] which is attributable, at least in part, to their effects on platelets. Specifically, a supplementation for 12 weeks with anthocyanins, natural plant pigments with a wide range of biological activities and cardiovascular protective activity, significantly improves cholesterol efflux capacity [237] and anti-oxidative and anti-inflammatory capacity [237], associated with positive modulating effects on platelet function [238]. Benefits from anthocyanin administration were also observed in vitro, in mice fed high-fat diets [239,240,241], and in patients with hypercholesterolemia where the supplementation of anthocyanins inhibited platelet granule release [242]. A linear dose-dependent effect of anthocyanins on reducing platelet activation, aggregation, and oxidative stress biomarkers has been recently found after a 12-week anthocyanin intervention in patients with dyslipidemia [243]. In particular, the treatment of dyslipidemic patients with anthocyanins reduced platelet levels of ROS and urinary concentrations of the in vivo stress oxidative marker 8-iso-PGF2α, which positively correlated with the attenuation of platelet hyperreactivity [243].

A gene expression analysis revealed that antioxidants can down-regulate PCSK9 and upregulate LDLR expression by activating AMPK, which in turn influences the expression of PCSK9 and LDLR. The improvement of the lipid profile and the reduction in body weight in obese mice were particularly observed after treatment with naringin (4′,5,7-trihydroxy flavanone 7-rhamnoglucoside), a flavonoid coming under the subclass of flavonones [244] which is commonly found in citrus fruits and is responsible for their bitter taste [245]. The protective role of naringin on platelet function may be ascribed to its antioxidant (ROS/RNS pathways), anti-inflammatory (COX-2, IL-6, TNF-α, NF-κB pathways) properties and its ability to modulate PCSK9 [246].

## 9. Conclusions

Hypercholesterolemia is a well-recognized adverse factor closely implicated in the increased risk of CV disease, and this link is supported by the efficacy of LDL-lowering therapies in reducing the incidence of fatal and non-fatal atherothrombotic events. This prothrombotic phenotype is specifically triggered by the action of oxLDL, the product of dysfunctional lipid metabolism, on platelet function as a result of exacerbated ROS production or their insufficient scavenging. The measurement of different biomarkers of redox function shows that, in parallel to increased products of oxidative stress such as ROS, peroxynitrite, TXB2, and isoprostanes, a further driver of this redox disequilibrium is the reduction of enzymatic and non-enzymatic antioxidant defenses such as GSH, SOD and CAT levels. Indeed, lipid-lowering agents such as statins and PCSK9 inhibitors have been demonstrated to improve clinical outcomes not only for their action in improving the cholesterol profile but also for their antithrombotic properties which include their common ability, in platelets, to positively interfere with the imbalanced redox status.

To conclude, even though platelets are the smallest blood cells, monitoring platelet biomarkers of oxidative stress and antioxidant status could help to assess the big problem of prothrombotic tendency for patients at high CV risk.
